# Diagnostic Abilities for Determining the Level of Blood Cryoglobulins in the Choice of Tactics for Operations on the Small Intestine

**DOI:** 10.25122/jml-2020-0083

**Published:** 2020

**Authors:** Svetlana Mykolayivna Gramatiuk, Irina Yurievna Bagmut, Julia Viktorivna Ivanova, Mykhailo Yevhenovych Tymchenko, Igor Vadimovich Kryvorotko, Svetlana Oleksyiyivna Pak, Michael Ivanovich Sheremet

**Affiliations:** 1. Department of Clinical Research, Ukraine Association of Biobank, Kharkiv, Ukraine; 2. Department of Clinical Pathophysiology, Topographic Anatomy and Operative Surgery, Kharkiv Medical Academy of Postgraduate Education, Kharkiv, Ukraine; 3. Surgery Department No. 1, Institute of General and Urgent Surgery of V.T. Zaytsev National Academy of Medical Sciences of Ukraine, Kharkiv, Ukraine; 4. Department of Perinatology, Obstetrics and Gynecology, Kharkiv Medical Academy of Postgraduate Education, Kharkiv, Ukraine; 5. Surgery Department No. 1, Bukovinian State Medical University, Chernivtsi, Ukraine

**Keywords:** Cryoglobulinemia, anastomotic leak, small intestine anastomoses

## Abstract

The study of the incidence of cryoglobulinemia is relevant in patients with an intestinal anastomotic leak. This study aims to determine a laboratory marker of the risk of small intestine anastomotic leak. The study was based on 96 patients who were subjected to resections of segments of the small intestine with the formation of intestinal anastomoses at the State Institution “Zaytsev V.T. Institute of General and Urgent Surgery of National Academy of Medical Sciences of Ukraine”. Of all the operated patients, there were 55.2% women and 44.8% men. Of the 96 patients examined, cryoglobulinemia was detected in the majority – 62.5% of patients, of which 4 were later proved to have inactive hepatitis C; the remaining 38.5% had no cryoglobulinemia. According to the existing theory of the autoimmune mechanism of postoperative surgical complications formation, the revealed decrease in the level of cryoglobulins on the second day could be related to their fixation in the microcirculatory bed and the development of immunocomplex inflammation. While the increase in the content of cryoglobulins in serum on the third day can be caused by their entry into the circulatory bed from deposition or fixation sites and the development of a secondary immune response. In patients with intestinal anastomosis failure after resection of intestinal segments, cryoglobulinemia rates increased more than 80 mg/l; this indicator could be used as a marker of postoperative complications.

## Introduction

Bowel resection is the most frequent operation which is performed according to urgent indications [1-4]. It is known that the leading pathophysiological aspect in patients with intestinal anastomosis is volemic and hemodynamic disorders, which are caused by the reduction of arterial inflow and impaired venous outflow due to compression of the intracellular vessels and fluid sequestration. In 15-25% of cases, the course of urgent surgical diseases is complicated by peritonitis. According to different authors, mortality rates range from 10 to 60% in common forms of peritonitis, and in-hospital mortality rates of peritonitis can reach 90% [5–9].

The primary source of endotoxicosis in patients with intestinal anastomosis failure is the intestine [10–15]. Violation of the intestinal wall’s barrier function leads to endotoxemia, which in the absence of adequate treatment is progressive [16–18].

The generalization of damaging effects is mediated by the widespread prevalence of TNF receptors and other cytokines’ ability to liberalize it. From a practical point of view, it is extremely important to note that the reaction rate of the septic cascade in widespread peritonitis increases sharply under conditions of hypoxia due to the expression of cytokine receptors on the cell surface [19 – 24].

In our opinion, and this is confirmed by studies in related pathologies [25, 26], it is relevant to study the incidence of cryoglobulinemia in patients with cancer insolvency, which is appropriate in the aspect of prevention of postoperative complications, rapid recovery of bowel functions and further effective rehabilitation of operated patients.

The aim of the study is to determine a laboratory marker of the risk of small intestine anastomotic leak.

## Material and Methods

The study was based on the results of laboratory studies of 96 patients who were subjected to resections of segments of the small intestine with the formation of intestinal anastomoses at the State Institution “Zaytsev V.T. Institute of General and Urgent Surgery of National Academy of Medical Sciences of Ukraine” between 2017 and 2019. The mean age of the patients was 54.7±5.9 years. Of all the operated patients, there were 53 (55.2%) women and 43 (44.8%) men. Patients were screened according to criteria, and those who were divided into study groups had small intestinal anastomosis, and a score from 5 to 25 on the Acute Physiology and Chronic Health Evaluation (APACHE II). Concomitant pathologies in cases that had postoperative complications (failure of anastomosis, peritonitis, wound healing failure, bowel necrosis, bleeding, and non-surgical complications) were also assessed. There were no statistical differences among any groups with respect to the by concomitant pathology.

This is due to the fact that the previous data did not show a significant difference between the groups with intraoperative complications, p≥0.05, and did not reveal a probable difference in the groups without intraoperative complications. Therefore, to further study the condition of patients, we identified two groups of patients: with and without cryoglobulinemia. Patients were divided into two groups: group I consisted of patients with uncomplicated postoperative period, group II - patients with a complicated course of the postoperative period (accounted primarily for purulent-inflammatory surgical complications). The control group consisted of 25 conditionally healthy persons (men and women) of the same age (from 35 to 66 years).

Cryoglobulins are serum immunoglobulins that re-precipitate at temperatures below 37°C. Separation of cryoglobulins from the serum was performed by the A.E. Kalovidoris method with modifications. The concentration of cryoglobulins was evaluated on the SF-46 spectrophotometer in dynamics on the first, second, third, and seventh day. Control values for cryoglobulins in serum were tested in 15 healthy subjects and ranged from 60 to 80 mcg/ml, which corresponds to the norms obtained by Ferri et al. in 2002 [27]. Study of the total population of T-lymphocytes (CD3+), subpopulations of T-lymphocytes - T-helper (CD4+), T-suppressors (CD8+) and β-lymphocytes (CD19+ and CD20+) in serum was performed using monoclonal antibodies - CD4+, CD8+, CD19+ and CD20+ by the immunofluorescence method on the “STAT-FAX303” (USA) enzyme-linked analyzer. The content of immunoglobulins A, M, G (IgA, IgM, IgG), and total immunoglobulin E (IgE) in the serum was examined using enzyme-linked test systems of the production of “Granum-Ukraine” (Ukraine), and the content of allergen-specific IgE was investigated using enzyme-linked immunosorbent assay systems produced by “Microgen” (Russia). Studies of circulating immune complexes were determined in serum by the method of Gashkova et al., and serum tumor necrosis factor-alpha (TNF-α) was detected using enzyme-linked immunosorbent assay systems manufactured by “Protein Contour” (Russia) and “Diaclone” (France).

Statistical analysis was performed using the Statistica 6.0 software (Stat Soft, Inc. 2001) and SPSS 7.5 on an Apple PC.

## Results

**Figure 1:**
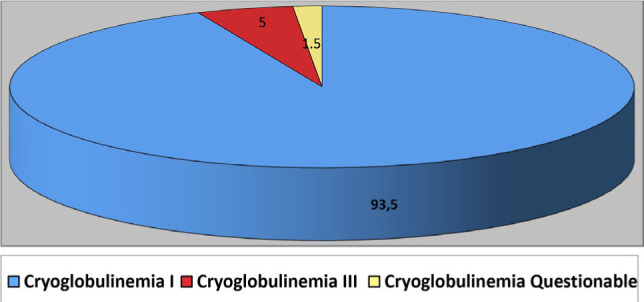
Distribution of patients according to the classification of cryoglobulinemia type by J. S. Brouet.

Of the 96 patients examined, cryoglobulinemia was detected in the majority – 59 (62.5%) patients, of which 4 were later proved to have inactive hepatitis C; the remaining 37 patients (38.5%) had no cryoglobulinemia. Because cryoglobulinemia is not the only sign, in our study, we used the classification of J. S. Brouet (1974) [28]. According to this classification, cryoglobulins were divided into three types, depending on the components included in the cryoprecipitate [29, 30]. The first type includes immunoglobulins of the same class with one type of light chain (IgG, IgM and IgA). The second type is mixed cryoglobulins, which are combined with second class polyclonal Ig. The monoclonal component of this class has rheumatoid factor activity and is IgM. The third type includes mixed cryoglobulins containing complexes of polyclonal immunoglobulins IgG + IgM, IgG + IgM + IgA, as well as IgM with low molecular weight. In our study, 95% of patients had the first type of cryoglobulinemia that we associate with self-aggregation through the Ig Fc fragment, and 5% had the third type of cryoglobulinemia that is related to the latent form of hepatitis C in these patients (Figure 1).

We first conducted a study of the quantitative content of cryoglobulins in patients who underwent resection of the small intestine segments with the formation of intestinal anastomoses. As a result of the obtained data, we made the distribution of patients according to the degree of cryoglobulinemia: low, medium, and high (Table 1).

**Table 1:** Distribution of patients who underwent resection of the segments of the small intestine with the formation of intestinal anastomoses by the degree and type of cryoglobulinemia.

**Severity of cryoglobulinemia**	**Type of cryoglobulinemia**		
	**Cryoglobulinemia I (n=53)**	**Cryoglobulinemia III (n=4)**	**Cryoglobulinemia questionable (n=2)**
**Low cryoglobulinemia (mg/l)**	79.4 ± 1.01	82.3 ± 1.05	-
**Moderate cryoglobulinemia (mg/l)**	298.6 ± 2.5**	325.1 ± 2.2**	315.1 ± 2.0**
**High cryoglobulinemia (mg/l)**	450.5 ± 12.4**	477.3 ± 10.0**	496.5 ± 8.7**

Note: * p<0.05 relative to normal values, ** P<0.05 relative to between groups.

Most patients who underwent resection of segments of the small intestine had type I of cryoglobulinemia and the content of cryoglobulins was low and medium (53 patients); patients with questionable cryoglobulinemia did not have a low cryoglobulin content. However, this may be due to the small number of patients in this group (2 patients). As a result of the study, it was found that all examined patients had a significant increase in cryoglobulin levels by an average of 106.5% on the first day after surgery. It should be noted that high- and moderate-grade cryoglobulinemia was observed in patients whose postoperative period was further complicated by the development of postoperative complications.

**Figure 2:**
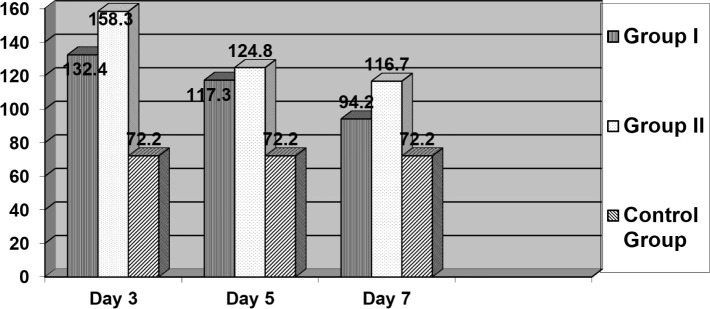
Dynamics of immunoglobin content in cryoprecipitate in the studied patients (n=96).

We first investigated the content of cryoprecipitates in patients with the inefficiency of intestinal anastomosis in parallel with the concentration of immunoglobulins G and M, as well as immunoglobulins A. The dynamic study of the content of cryo-complexes in the investigated patients established a decrease in Ig pathogens, anastomosis associated with fixation of cryoglobulins in the microcirculatory bed with manifestations of autoimmune aggression with respect to the gut epithelium. On the seventh day of the disease, an increase in IgG content of 4.3% and a significant (p≤0.05) increase in IgM by 19.4% relative to the initial level were established. Increasing the content of antibodies at day seven may be associated with the development of a secondary immune response. In some cases, the increase of IgG and IgM concentration relative to their initial level occurred as early as the third or fifth day of observation (Figure 2).

In both groups of patients, a decrease in the concentration of cryoglobulins was found on the second day after surgery and a significant increase on the third day. On the fifth day, there was a decrease in the concentration of cryo-proteins in the blood of patients from 158.3±28.6 mcg/ml to 124.8±10.8 mcg/ml in group II. Also, a reduction from 132.4±82.2 mcg/ml to 117.3±63.9 mcg/ml was noted in group I.

When studying the initial parameters of the immune status in patients of the studied groups, before surgery and on the first day after surgery, no significant differences were noted (p>0.9). However, it was found that in patients with cryoglobulinemia, the immunogram values were significantly lower (p≤0.05).

In the peripheral blood of patients with cryoglobulinemia, there was a significant decrease in the relative number of CD3+ relative to the comparison group (66.2±0.5%, p<0.05). However, with the uncomplicated course of the postoperative period, this index practically approached normal values (63.47±0.68%). In contrast, in the second group, until the end of treatment, there was a tendency to increase the blood content of CD3+ (55.53+0.65%). The results of the study of subpopulations of lymphocytes are presented in Table 2.

**Table 2:** Dynamics of the content of lymphocyte subpopulations in the studied patients.

**Blood count**	**Norm**	**Group 1 (n=59)**		**Group 2 (n=37)**	
		**Day 1**	**Day 5**	**Day 1**	**Day 5**
**CD3+(T-lymphocytes), %**	66.2 ± 0.5	47.12 ± 0.91*	63.7 ± 0.68	50.93 ± 0.83*	55.53 ± 0.65**
**CD4+(T-helpers), %**	43.9 ± 0.8	29.12 ± 0.61*	40.51 ± 0.46	30.77 ± 0.6*	33.3 ± 0.66**
**CD8+(T-cytotoxic), %**	27.0 ± 0.9	15.21 ± 0.35*	22.11 ± 0.5	16.2 ± 0.3*	18.45 ± 0.41
**CD16+(NК–cells), %**	13.5 ± 0.7	19.12 ± 0.45*	12.56 ± 0.34	18.97 ± 0.41*	14.73 ± 0.32
**CD 20+(B-lymphocytes), %**	14.0 ± 0.2	27.4 ± 0.27*	22.6 ± 0.29	27.67 ± 0.25*	25.67 ± 0.37**
**CD4+/CD8+**	1.9 ± 0.02	1.92 ± 0.18	1.86 ± 0.15	1.87 ± 0.16	1.7 ± 0.15

Note: * p<0.05 relative to normal values, ** p<0.05 relative to group I.

The level of CD4+ and CD8+ was also significantly reduced compared to group I: on the fifth postoperative day, the level of CD4+ in patients of group I increased to 40.51±0.46%, compared to 33.3±0.66% in group II (p<0.05).

In addition, a significant (p<0.05) increase in the level of NK cells was found, which may be associated with impaired barrier function of the intestinal mucosa and penetration into the submucosal layer of antigens of the intestinal flora.

When determining the level of CD20+ in patients of group I, it was revealed almost a twofold increase in this indicator relative to group II, so in patients with cryoglobulinemia, this indicator was (27.4±0.27%). In patients with an uncomplicated postoperative period, a more pronounced tendency of the CD20+ level normalization was observed in patients of group I compared to patients of the control group (p<0.05).

Patients in group I showed a 2-fold increase in IgA levels (5.11±0.07 g/l) at a normal rate of 2.5±0.08 g/l, due to the fact that IgA immunoglobulins are the “first line of defense of the organism” in the mucous membranes of the gastrointestinal tract. When studying the level of immunoglobulins of IgM and IgG classes, significant (p<0.05) deviations from the normal parameters were established (Table 3).

**Table 3:** Level of IgA, IgM and IgG in patients in study groups.

**Blood count**	**Norm**	**Group I (n=59)**		**Group II (n=37)**	
		**Pre-treatment**	**Day 5**	**Pre-treatment**	**Day 5**
**Ig A, g/l**	2.5 ± 0.08	5.11 ± 0.07*	3.75 ± 0.06	4.85 ± 0.07*	4.27 ± 0.05**
**Ig M, g/l**	1.51 ± 0.05	1.18 ± 0.05	1.30 ± 0.04	1.22 ± 0.05	1.25 ± 0.04
**Ig G, g/l**	14.7 ± 0.42	16.3 ± 0.2	14.84 ± 0.10	15.55 ± 0.07	15.17 ± 0.09

Note: * p<0.05 relative to normal values, ** p<0.05 relative to group I.

The examined patients revealed a significant decrease in the phagocytic activity of neutrophils and phagocytic number, regardless of the presence of cryoglobulinemia.

More rapid and complete recovery of the phagocytic activity (64.2±1.65%) and phagocytic number (4.72±0.11%) occurred in group I compared with group II (53.03±1.03% and 3.85±0.1%, respectively), which created the prerequisites for the restoration of phagocytosis and its completion, and, consequently, the reduction of the risk of complications in the postoperative period (Table 4).

**Table 4:** Phagocytic activity of neutrophils in test patients depending on cryoglobulinemia.

Blood count	Norm	Group I (n=59)		Group II (n=37)	
		Day 1, %	Day 5, %	Day 1, %	Day 5, %
**Phagocytic activity of neutrophils, %**	66.32 ± 2.11	46.68 ± 1.14*	64.2 ± 1.65	45.23 ± 0.89*	53.03 ± 1.03**
**Phagocytic index**	5.5 ± 0.4	2.77 ± 0.05*	4.72 ± 0.11	2.80 ± 0.06*	3.85 ± 0.1**

Note: * p<0.05 relative to normal values, ** p<0.05 relative to group I.

Thus, the conducted studies have established that in patients with cryoglobulinemia, who suffered resections of the bowel segments, a secondary immune deficiency is formed, determined by abnormalities in the cellular and humoral immunity systems.

Earlier and more complete restoration of immune status indicators occurs in patients with cryoglobulinemia, in which the postoperative period proceeded without complications.

**Figure 3:**
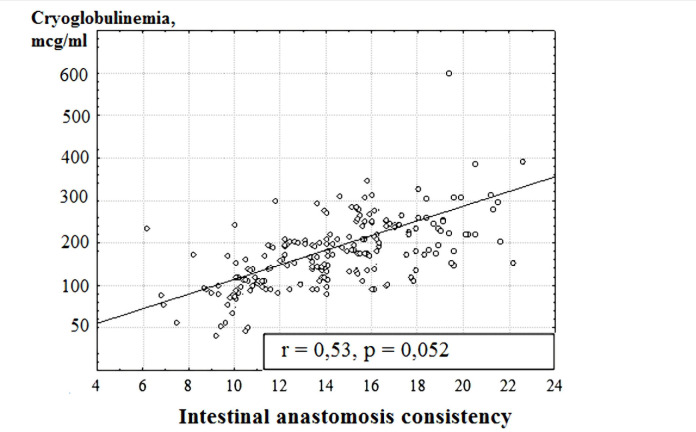
The correlation between the initial level of cryoglobulins and the intestinal anastomosis consistency.

The conducted correlation analysis revealed a direct correlation between the initial content of cryoglobulins and the ability of intestinal anastomoses (r=0.56, p=0.07, and r=0.53, p=0.052). Detection of high concentrations of cryoglobulins in the serum of operated patients in the first hours revealed the possibility of maximal autoimmune changes during the development of purulent-inflammatory complications (Figure 3).

In both groups of patients, a decrease in the concentration of cryoglobulins was found on the second day after surgery and their significant increase on the third day. On the fifth day, there was a decrease in the concentration of cryo-proteins in the blood of patients from 158.3±23.6 mcg/ml to 114.8±10.8 mcg/ml in the group with anastomotic failure and from 132.4±12.2 mcg/ml to 97.3±13.9 mcg/ml in the group with an uncomplicated postoperative period.

## Discussion

According to the existing theory of the autoimmune mechanism of postoperative surgical complications formation, the revealed decrease in the level of cryoglobulins on the second day could be related to their fixation in the microcirculatory bed and the development of immunocomplex inflammation [31, 32]. In contrast, the increase in the content of cryoglobulins in serum on the third day can be caused by their entry into the circulatory bed from the sites of deposition or fixation, and the development of a secondary immune response [33–35].

This study found a significant difference (p≤0.05) between the concentration of cryoglobulins and the presence of postoperative complications in the studied patients. The available cryoglobulin concentrations of 162.32.6 mcg/ml were observed in patients with postoperative complications directly related to surgery. Until the end of observation in patients with an uncomplicated postoperative period, a significant decrease in cryoglobulin concentration was found. In this group of patients, the serum cryoglobulin level on the seventh day of the postoperative period approached normal values (60-80 mcg/ml) and averaged 75.7 mcg/ml. The obtained data indicate the effective elimination of cryoglobulins from the body of patients with an uncomplicated postoperative period [36]. The decrease in the level of cryoglobulinemia in this group was correlated with the disease course, improvement of the postoperative wound condition, and normalization of rheological parameters of the blood. Also noteworthy is the lack of lethality in the group of patients with normal cryoglobulins.

The dynamic study of the composition of cryo-complexes in operated patients revealed a decrease in the content of IgG and IgM, which is associated with the fixation of cryoglobulins in the microcirculatory bed with the manifestation of autoimmune aggression against the intestinal epithelium. On the seventh day of the disease, an increase in the IgG content by 4.3% and a significant increase in the IgM level by 19.4% from baseline were found. An increase in antibody content on day seven may be associated with the development of a secondary immune response. In some cases, the increase in the concentration of IgG and IgM relative to their level on the second day occurred already before the third and fifth day of observation.

A decrease of 30.3% for IgM and 19.5% for IgG was found in a short period after surgery, which was significantly correlated with a reduction in the concentration of cryo-proteins in the circulation (r=0.95 for IgG and r=0.98 for IgM, p<0.05). This was accompanied by an improvement in the patients’ general condition, an increase in the clinical score on the Scale for the Assessment of Positive Symptoms (SAPS) scale (r=-0.6; p<0.05). The results obtained by us on the dynamics of cryoglobulin change in patients with surgery on the small intestine make it possible to assume their considerable heterogeneity in properties.

Thus, the revealed changes in the content of immunoglobulins G and M (increased concentration of IgM in the fifth day of the disease, and IgG in the seventh day) suggest that the development of the mechanism of cryoglobulinemia in operated patients is similar to the mechanism of development of the primary immune response. The presence of an initially increased number of cryo-proteins in the intestinal obstruction may be higher due to the processes of thrombosis and thrombolysis that is constantly occurring in the body of such patients, and, consequently, hyperactivity of the coagulation and anti-coagulation systems of the blood [37–39]. In the group with an uncomplicated postoperative period, the reduction of cryoglobulinemia as a result of the therapy was determined on the seventh day of observation, which was correlated with some improvement of the severity of the condition (on the SAPS scale) of patients by the end of the first week (r=-0.49, p<0.03), which correlated with significantly lower SAPS scores. This study revealed a significant effect of the postoperative period on the level of decrease in the viscosity of whole blood by the end of the postoperative period in patients with an uncomplicated period - in 93.6% of patients with an average of 76.3±12.02% with a reliability of p<0.05 compared to group II with a complicated postoperative period.

## Conclusion

In patients with intestinal anastomosis failure after resection of intestinal segments, cryoglobulinemia rates increased more than 80 mg/l. Therefore, this indicator could be used as a marker of postoperative complications. The analysis revealed a direct correlation between the content of cryoglobulins and the ability of intestinal anastomoses, which allowed using these indicators as the most informative criterion for the prediction of postoperative complications.

## Conflict of Interest

The authors declare that there is no conflict of interest.
